# Exploratory study of the characteristics of feedback in the reflective dialogue group given to medical students in a clinical clerkship

**DOI:** 10.3402/meo.v20.25965

**Published:** 2015-02-06

**Authors:** Chin-Chen Wen, Meei-Ju Lin, Chi-Wei Lin, Shao-Yin Chu

**Affiliations:** 1 Department of Human Development, Tzu Chi University, Hualien, Taiwan; 2 Department of Counseling & Clinical Psychology, Dong Hwa University, Hualien, Taiwan; 3 Department of Paediatrics, Buddhist Tzu Chi General Hospital, Hualien, Taiwan; 4 Department of Medicine, College of Medicine, Tzu Chi University, Hualien, Taiwan

**Keywords:** feedback, reflective dialogue group, undergraduate medical education

## Abstract

**Purpose:**

Structured narrative reflective writing combined with guided feedback is an efficient teaching method for enhancing medical students’ reflective capacity. However, what kinds of feedback offered and reflection presented in a reflective group remain unclear. The aim of this study was to investigate the characteristics of feedback in a reflective dialogue group.

**Methods:**

Fifth-year medical students on a monthly interval rotation at the pediatric department of a medical center in eastern Taiwan during the 2012 academic year completed their reflective writing regarding patient and family psychosocial issues, and were subsequently debriefed in a 2-h group discussion session to receive feedback from a clinical tutor and peers. Content analysis was conducted to explore the characteristics of feedback and reflection presented in the reflective dialogue. The evaluative questionnaire regarding the benefits of reflection with others was administrated following the group session.

**Results:**

Forty students participated in five reflective groups and 108 psychosocial issues were discussed and identified. The tutor played an initiating role in the group discussion by providing six equal feedback types involving exploring new knowledge, initiating advanced discussion, highlighting the issues, and encouraging the students. The students provided eight types of feedback that involved a substantial deep discussion on psychosocial issues and action plans based on the complex interactive ecological network of clinical encounters. Each student attained 1.25 times the depth or breadth of reflection after receiving feedback and experienced the benefits of reflection with others.

**Conclusion:**

Through structured narrative reflective writing combined with pluralistic group discussion with a tutor and peers, the medical students had time to think deeply and broadly about psychosocial issues among patients and their family members. Facilitative feedback providing new knowledge, deeper discussion, and exploring new ways of action planning for psychosocial issues was recommended to promote students’ reflective capacity.

A reflective practitioner and reflective rationality involve professionals bridging the gap between theory and practice, with an attitude toward working from experience by narrating, rethinking, and reframing this gap (1). Reflection can occur in three moments: reflection-in-action, reflection-on-action, and reflection-for-action. Therefore, reflection provides professionals an opportunity to think broadly and deeply about their practice, and reflective capacity has been incorporated into one of the professional competencies of an effective physician (2). Charon studied physicians who used a reflective narrative regarding stories of patients, themselves, colleagues, and society for enhancing respectful, empathic, and nurturing medical care (3).

The use of reflection in medical education refers to three teaching methods (4–9). First, medical educators could apply a reflective model, such as Gibbs’ reflective cycle (10), to construct reflective questions to facilitate broad and deep reflections. Second, most reflection involves reflection-on-action after the event has occurred in medical practice; thus reflective writing, such as an unstructured diary or a guided reflective journal, could recapture patient–physician encounters in elaborative, analytic approaches for transforming the experience further. Finally, guided reflection by a faculty mentor or peer group members encourages people to share their thoughts, feelings, or reactions to their experiences reciprocally, and promotes multiple perspectives and alternative actions.

Medical curricula that apply these teaching methods to facilitate medical student reflection have been widely developed and effectively examined. DasGupta and Charon (11) designed a 6-week seminar of reflective writing for 16 second-year medical students to describe their own illness story, or that of a family member or friend. The written essays were shared and discussed each week, and qualitative evaluations at the final session showed increasing amounts of student empathy and self-awareness. The other qualitative study combined structured reflective writing of field notes and individualized faculty feedback for preclinical students in a ‘doctoring’ course for first- and second-year students. The thematic qualitative evaluations of field notes and feedback showed the following advantages: deep reflection, valued feedback, promoting later group process, and personal and professional development (12). A randomized comparison study examined the teaching effectiveness of guided critical reflection and faculty feedback for 149 third-year students. One question about reflective ability was evaluated and indicated that the teaching methods of either critical reflection guidelines or feedback on the reflective process have more benefits for improving student reflective ability than teaching without any guideline or feedback (13). Another study compared two teaching designs, the traditional written format with a small-group discussion, and the blog format following an online commentary and feedback for clerkships, and found that the level of reflection did not differ between the two teaching designs, whereas students’ preferences for blogging or essay writing influenced their learning experience (14).

Both qualitative and quantitative studies have indicated that combined structured reflective writing and guided feedback from a mentor or a group is the most efficient teaching method for enhancing student reflective capacity (11–14). Theories have also suggested that group work or one-to-one discussions facilitate the later stage of the reflective learning cycle; however, few studies have specifically explored the characteristics of effective feedback (15). Sandars indicated that the role of the facilitator during feedback should include counseling and mentoring skills for building a supportive environment to enable students to process the reflection (5). Branch and Paranjape addressed the general rule that facilitating reflection involves elaborating on a topic and transferring it to a deeper or more complex level of reflection (16). The initial study found that elaborate questions and a positive tone were effective writing-feedback comments to stimulate student reflection (17). However, what kinds of feedback offered and reflection presented in a reflective group remains unclear.

In this study, we conducted content analysis to explore the characteristics of feedback and reflection presented in the reflective dialogue group composed of a clinical tutor and medical students during their clinical clerkship in a pediatric department. The following research questions were addressed: What were the characteristics of feedback provided from clinical mentor and students in the reflective dialogue group?; Did medical students think broader and deeper about their practice after receiving feedback from tutor and peer?; and, How did medical students perceive the benefits after the reflective group session.

## Methods

### Study background and procedures

This study was implemented in a required clinical course that involved writing about patient and family psychosocial issues, designed for fifth-year medical students on a monthly interval rotation (7–9 students on each rotation) at the pediatric department of a medical center in eastern Taiwan during the 2012 academic year. All 50 students planned to rotate at the pediatric department, completed the reflective writing, and participated in one of the six group sessions separately in 2012.

During the process of primary care and history taking interviews in each rotation, each student separately chose and observed the psychosocial issues of one patient and his family members, completed their reflective writing at the third week of rotation, and subsequently discussed in a 2-h group session to receive feedback from the course tutor and peers. The reflective writing was structured by guidelines developed from Gibbs’ concept of reflective cycle (10), which comprises six components: description, feelings, evaluation, analysis, conclusion, and action plan.

During the reflective group section, 7–9 medical students sat in a circle and received a short orientation regarding the group activity process. Each debriefed student demonstrated psychological issues in a reflective approach, and received one peer feedback that particularly focused on alternative action plans. Tutor feedback was given using reinforcement teaching skills, the sandwich feedback method, and possible alternative approaches for solving identified psychosocial issues. Following group session, the students were asked to complete an evaluative questionnaire about the benefits of reflection with others.

This study was conducted after obtaining approval through informed consent from the institutional review board at Buddhist Tzu Chi General Hospital (IRB102-20). The group was designed and led by the corresponding author. Group sessions were recorded by a tape recorder after receiving consent from the students. In addition to the instructor, at least one member from the research team joined the group dialogue session and observed the whole process.

### Data analysis

#### Group process analysis

All group sessions were anonymously transcribed by two research assistants. Each session was subsequently divided into three analyzed parts: characteristics of feedback, dialogues involving considerable depth or breadth of reflection, and psychosocial issues discussed during the session.

Content analysis is a method used to deal primarily with verbal materials to extract desired information or identify specific characteristics through a multistep process. The advantage of the content analysis lies in its ability to transform the qualitative information into quantitative information such as category frequencies or ratings for further comparison (18). In the present study, we applied content analysis to identify and code the characteristics and frequencies of feedback, and dialogues involving considerable depth or breadth of reflection in the reflective dialogue group.

First, the research team undertook a trial analysis on one group's transcript and found certain types of feedback emerged consistently as to develop a preliminary coding list of feedback. We then used this preliminary coding list to code the second group transcription until the taxonomy of feedback had been discussed elaborately and fully. As a result, we defined eight types of feedback between the students, and six types of feedback from the tutor to the students (Appendices 1 and 2). Second, we developed the coding manual regarding the dialogues involving depth or breadth of reflection based on the Mezirow theory (19). We defined ‘the depth of reflection’ as the presence of analyzing, exploring, and reflecting all assumptions or problem-solving strategies regarding psychosocial issues or action plans. ‘The breadth of reflection’ was defined as the presence of discussing derived topics related to psychosocial issues or action plans. Third, two researchers as a team coded the last group materials separately by using the coding manual and then discussed the codes consensually. If there was a disagreement, the third researcher was consulted to reach an agreement.

All psychosocial issues discussed during the group sessions were openly reviewed and identified, and named consensually by researchers. The frequencies of psychosocial issue, feedback, and the depth or breadth of reflection were calculated as category frequencies.

#### The evaluative questionnaire

Jasper stated reflective practice has many advantages for professional development especially by using reflective strategies with others (20). We further conducted a 10-item evaluative questionnaire, with items such as ‘They provide me with immediate feedback’, with a five-point scale to measure the degree of agreement on the reflective benefits with peers evaluated by students, where 1 stood for strongly disagree and 5 stood for strongly agree.

The chi-square test was used to analyze the frequency and differences of feedback types and the depth or breadth of reflection. The mean and standard deviation of the 10 items of the evaluative questionnaire were also statistically analyzed. All data were computed using the Statistical Package for Social Science, version 15.0 (SPSS Inc., Chicago, IL, USA).

## Results

Except for one piloting group, this study analyzed the reflection dialogue process and evaluative questionnaires of five group sessions including 40 medical students. Male to female ratio was 24:16, and the mean age was 24.7 years old. There were 108 psychosocial issues identified from five group coding transcripts which categorized into 7 major themes, including medical communication, the intricate medical ecological system, role and function of a family, medical students’ competence toward a medical profession, ethical dilemma in clinical setting, and extreme diverse patient perspectives from different sociocultural background underlying the complexity of clinical encounter. One sample case demonstrated how student A debriefed controversial psychological issues involving young adolescents, dropping out of school and family, misconduct, and economic problems along with group dialogues.

One 17-year-old girl dropped out from high school working as a waiter at a night shop for more than one year, admitted due to dysuria and lower abdominal pain. She had a history of artificial abortion recently; urinary tract infection and pelvic inflammatory disease were diagnosed this time. None of her family members visit her during the whole admission course except her boyfriend and the boss of the night shop. I think she must come from low social economic status, facing difficulties in schooling, poor family relationship, struggling for living and with behavioral problems. She may encounter bad friends, be exploited, engaged in sexual trades, controlled by drugs, and unable to appeal her hell … My difficulties were: I have so different growing environment as compare to patient. I can't seem to understand this girl's living environment and her thoughts. I don't know how to communicate with her in the first setting. I am afraid that I may hurt her unintentionally. And most importantly, I don't know how to stand for her and change this complex situation ….

### Group dialogue

Phrases in parentheses are types of feedback identified.

Feedback from students (Deeply discussing psychosocial issues)

**Table:** 

Student B	Can you concise the psychosocial issue?
Student A	It was so complicated …. She had a lot of risk factors ….
Student B	What is the major problem you want to change?
Student A	I think the way to prevent another episode of urinary tract infection through changing those risk behaviors is very important

Feedback from the tutor and students

**Table:** 

Tutor	Young adolescents drop out from school, generation gap among parents and children, legal and ethical issues on artificial abortions were clearly addressed psychosocial issues. Did you see the issue related to child neglect? (Expend and diverse to other issues)
Student A	It was very hard to approach the issues upon artificial abortion; we need to learn and try more ….
Student D	Is working at night shop a psychosocial issue addressed? Creating another working opportunity out of night shop may be one way to solve her problem …. (Confronting or debating psychosocial issues)
Tutor	What is your impression on night shop? (Deeply discussing psychosocial issues)
Student A	The environment is not good for young adolescence's growth … it may relate to bad behavior ….
Tutor	Are we discussion about the issue of stigmatization? How about the public impression to a night shop? Please think about the role of us; as a physician, we always try to identify our patient into different risk groups in order to make a better differential diagnosis, are there any differences? (Explore and facilitate the discussion to cultural perspectives)
Student D	There are many kinds of night shop … the influences may not be all negatives … since we don't know exactly … the concern is that the living style is not healthy … encourage patient go back to school for study should also be tried. (Offering alternative courses of action)
Student C	The ecological status of the family should be evaluated and which may the leading cause of her problems …. (Offering alternative courses of action)
Tutor	Root cause analysis …. (Echoing the same experiences)
Student E	Why should she be study at school? We should not drive her future. What is her major interest should be concerned. The decision should be made by patient and her parent; we can only offer those options for her to make a better or may be suitable decision. (Confronting or debating action plans)

Some of the psychosocial issues were subject to cultural bounds, such as daughters or daughters-in-law in the families identified with traditional Taiwan or Chinese culture being expected to take care of ill parents, which usually makes them feel angry and frustrated. One student shared a story about a long-term care case of an old woman. While the son of the patient was given most of the inheritance of his father (the woman's deceased husband) and never showed up to visit the sick, her daughter took the duty of her care and complained about the unfairness. Both instructor and students in this study went onto discussing the male-preference phenomenon, and showed their sympathy for those daughters-in-law or daughters who took the most responsibility of patients’ care. Explanations of such psychosocial issues should take into account the sociocultural perspectives. Some other examples were shown as Appendices 1 and 2.

### Group feedback

The texts of five reflective dialogue groups were analyzed, with 107 types of feedback ‘from the tutor to the students’ and 73 ‘between the students’ (Table 1). Six types of feedback ‘from the tutor to the students’ were frequently categorized as follows: exploring new knowledge about psychosocial issues (25.2%), deeply discussing psychosocial issues (17.8%), exploring new knowledge regarding action plans (16.8%), deeply discussing action plans (15.9%), highlighting psychosocial issues (14%), and encouraging or approving students (10.3%). No significant differences were found between these six types of feedback (*X* ^2^ *=*7.90, *p*=0.16), indicating that tutor feedback was equally distributed among various categories.

Eight types of feedback ‘between students’ were frequently categorized as follows: deeply discussing psychosocial issues (50.7%), deeply discussing action plans (21.9%), confronting or debating action plans (8.2%), engaging in self-disclosure (6.8%), encouraging or approving (5.5%), confirming psychosocial issues (4.1%), confirming action plans (1.4%), and confronting or debating psychosocial issues (1.4%). Significant differences were found between these eight types of feedback (*X* ^2^=114.73, *p*=0.00), indicating that the feedback between the students in the reflective dialogue group was more centered on discussing psychosocial issues and action plans than the other categories.

### Depth and breadth of group reflection

Table 1 shows 50 dialogues coded as having the depth or breadth of reflection on psychosocial issues or action plans, meaning that each of the 40 students reported the psychosocial issues would have an average of 1.25 reflection statements that showed their increase in the depth or breadth of reflection after receiving feedback from the tutor and peers. These enriched or enlarged perspectives were shared in the group. No significant differences were found between these four levels of reflection (*X* ^2^=7.44*, p*=0.06); depth of reflection on psychosocial issues (*n=*20, 40.0%); depth of reflection on action plan (*n=*13, 26.0%); breadth of reflection on psychosocial issues (*n=*10, 20.0%); and breadth of reflection on action plan (*n=*7, 14.0%).

**Table 1:** Description statistical analysis of feedback, level of reflection

Feedback or level of reflection	Total, *n* (%)	*X* ^2^
Types of feedback between students	73 (100.0)	114.73*
Deeply discussing psychosocial issues	37 (50.7)	
Deeply discussing action plans	16 (21.9)	
Confronting or debating action plans	6 (8.2)	
Self-disclosure	5 (6.8)	
Encouraging or approving	4 (5.5)	
Confirming the psychosocial issue	3 (4.1)	
Confirming action plans	1 (1.4)	
Confronting or debating psychosocial issues	1 (1.4)	
Types of feedback from the tutor to students	107 (100.0)	7.90
Explore new knowledge about psychosocial issues	27 (25.2)	
Deeply discussing psychosocial issues	19 (17.8)	
Explore new knowledge about action plan	18 (16.8)	
Deeply discussing action plans	17 (15.9)	
Highlight the psychosocial issue	15 (14.0)	
Encouraging or approving students	11 (10.3)	
Level of reflection	50 (100.0)	7.44
The depth of reflection on the psychosocial issues	20 (40.0)	
The depth of reflection on action plan	13 (26.0)	
The breadth of reflection on the psychosocial issues	10 (20.0)	
The breadth of reflection on action plan	7 (14.0)	

* *p<*0.01.

### Evaluation of the benefits of reflection with others

Forty students evaluated the degree of agreement on the benefits of reflection in groups. The questionnaire showed good internal consistency (α=0.87). Two factor were extracted by exploratory factor analysis which contributing to 60.56% of the variance. Table 2 shows the students indicated averagely ‘agree’ to ‘strongly agree’ on the 10 benefits of reflection in groups (*M*=4.33, *SD*=0.16). There were no significant differences between these 10 items (*X* ^2^=1.2*, p*=0.99). However, the students indicated the highest agreement on the benefit of ‘They act as a sounding board for my ideas’ (*M*=4.6, *SD*=0.54).

**Table 2:** Evaluation of the benefits of reflection with others

Evaluative questions	Mean (SD)	*X* ^2^
They provide me immediate feedback	4.4 (0.53)	1.2
They help me to reflect on myself	4.4 (0.56)	
They act as a sounding board for my ideas	4.6 (0.54)	
They help me put events into a different context	4.4 (0.57)	
They offer a different way of seeing things	4.4 (0.61)	
They validate my thoughts about a situation	4.28 (0.67)	
Their feedback is a confirmation	4.1 (0.67)	
They bring their own knowledge and experience to the situation, which enhances my understanding	4.0 (0.64)	
They offer alternative courses of action about caring for patients or their families	4.38 (0.60)	
I can apply enhanced knowledge and understanding to the situation	4.36 (0.56)	
Total mean (SD)	4.33 (0.16)	

## Discussion

In this study, we designed a required course combining structured reflective writing and a reflective dialogue group to assist medical students in broadening and deepening their reflective thinking on psychological issues, further inducing a transformative action plan on humanistic caring.

The main results of this study showed how feedback operates and facilitates reflection on psychosocial issues and action plans from the tutor and peers. This study found that compared to student feedback, tutor feedback (107:73) tended to focus on varied feedback types, whereas the students learned to give limited types of feedback. Thus, the role difference derived from teaching and learning in reflective groups was salient. The tutor in this study was equipped with facilitative repertoires such as providing new knowledge or information, initiating discussion, highlighting topics, and encouraging the students. The tutor was particularly involved in sharing new knowledge, additional reading suggestions, and facilitating discussion from multiple perspectives about psychological issues, whereas the students were highly involved in acting as sounding boards and making meaning for other students. Positive feedback may prompt and facilitate building a topic focus and a friendly atmosphere to deepen the reflection (5, 16, 17).

As indicated, the role of the peer-assisted learning (PAL) phenomenon can be clearly observed in reflective dialogue-group dynamics (21). This study also found that most of the students were highly involved in deeply discussing psychosocial issues and action plans. Psychosocial issues reported by the students generally involved stories and controversial topics that caused them to realize during disclosure that they had similar reflections or faced similar clinical difficulties or complexities, which drew considerable attention and induced deep exploration (Appendices 1 and 2). The group also facilitated discussing an action plan and engaged in favorable exchanges of ideas regarding problem-solving and future implications. However, students engaged in few confrontations or debates with each other, which might be partially attributed to Chinese culture, which stresses humility and avoiding conflict. However, whether there was sufficient assertiveness in expressing opinions, particularly those that facilitate diversity, would be worth exploring. Thus, the long-term benefits and limitations of learning from unplanned PAL require further investigation.

Although there was no significant difference between the greater depth or breadth of reflection on psychosocial issues or action plans, this study found significant differences involving 66% of the reflection being related to ‘depth’ and 34% being related to ‘breadth’ (*X* ^2^ *=*5.12*, p=*0.02). In such a reflective group, greater depth either on psychosocial issues or action plans could be more easily addressed than the level of breadth on reflection. The reflective group enabled members to concentrate on the presented issues or actions and to respond with in-depth discussion. By contrast, although the discussion with increased breadth was less than in-depth, it still required a large proportion of time and should make effective contributions. However, this could be inferred only from cases in which the tutor provided equal feedback types and students were focused on a specific feedback type. Further study is necessary to confirm whether depth of reflection would be salient in other reflective groups, and to explore how to improve the breadth of reflection for medical students.

Reflection behaviors can be facilitated during group discussions. Each student attained 1.25 times greater depth or breadth of reflection after receiving feedback from the tutor and peers. Receiving feedback from the peers and tutor was also highly appreciated; the students indicated ‘agree’ to ‘very much agree’ on the benefits of reflection with others. The reflective dialogue group could be a welcome and effective course design for enhancing the reflection of medical students. While maintaining its effectiveness in increasing a discussion on psychosocial issues and action plans, facilitating optimal confrontation and debate through modeling or reinforcement may be necessary and worth attempting. Medical teachers should also assist students in organizing the perspectives generated in the group, regardless of the depth or breadth of consolidating their learning from peers and reflection.

The first limitation of this study is that we could not differentiate whether tutor or peer feedback contributes the greatest benefits and advanced level of reflection. Second, we could not infer whether the students showed more humanistic caring for patients and their families, as expected in the reflective course.

## Conclusion

Through structured narrative reflective writing combined with pluralistic group discussion with the tutor and peers, the medical students in this study had time to think deeply and broadly about psychosocial issues among patients and their family members. Facilitative feedback providing new knowledge, deeper discussion, and new ways of action planning for psychosocial issues was recommended to promote students’ reflective capacity.

## Appendix 1

Types of feedback between the students in the reflective dialogue group

**Table:**

Types of feedback	Description	Sample dialogues (indicating a psychosocial issue context)
Confirming the psychosocial issue	Validating the thoughts	A feedback is that you find that he is not easy to get close to after becoming acquainted with him, because I had the same experience. (Doctor–patient relationship: Alliance)
Confronting or debating psychosocial issues	Offering a different way of seeing things	If I were you, I would not mention these things to the patient's family, because this information was not approved by his attending physician. You would have a legal crisis of malpractice as a result of your conversation. (Medical communication and Professional roles: Worrying about whether a clerk has explained too much to a patient's family)
Deeply discussing psychosocial issues	Offering a sounding board for issues Providing the meaning of issues	It's an issue about ‘no filial long illness’. In the Taiwanese tradition of a patriarchal society, the eldest son or grandson typically inherits most of the family property. However, the daughter or daughter-in-law is assigned to take care of ill parents, which causes the women in this situation to become angry and frustrated. (Medical communication: Pointing out the sociocultural context) What is the value of a ‘doctor’? The most crucial ability is demonstrating humanity. The doctor should have positive interaction with people, care for them, listen to them, and exhibit courage. (Doctor–patient relationship)
Confirming action plans	Validating an action about a situation	I can try my best to listen to and accompany him. Because his mood is unstable, I try to remain positive. (Medical communication: Active listening when first meeting a patient with depression)
Confronting or debating action plans	Taking an objective stance about action	Do we really have the right or obligation to intervene in other people's lives to such a degree? (Doctor–patient relationship)
Deeply discussing action plans	Offering a sounding board for actions Offering alternative courses of action	Discussing action plans with patients’ family is general traditional in Taiwan, so the patient's family might accuse you of not been informed. (Medical communication about elderly population-conceal information: The role of the patient's family) When conducting health education, it is an advantage of indicating that a man who is an excessive smoker could experience sexual dysfunction. (Patient backgrounds: Education regarding poor family care)
Self-disclosure	Sharing the same difficulties Self-reflection	Regarding the issue of sex, we lack communication experience with young girls, and thus do not know how to discuss such a topic. (Patients’ backgrounds: Young adolescents with multiple sexual partners) When we first entered the clinic, our first priority was to focus on the disease, and then on the patient's history. We seldom care about the patient's family, but their body–mind–spirit also must be considered. (Grandparenting)
Encouraging or approving		However, the medical student excels in earning more patient trust than the attending physician does. (Medical communication: Worrying about whether a clerk has explained too much to a patient's family)

## Appendix 2

Types of feedback from the tutor to students in the reflective dialogue group

**Table:**

Types of feedback	Description	Sample conversations (indicating a psychosocial issue context)
Highlight the psychosocial issue	Strengthen and re-emphasis the psychosocial issues found Expend and diverse to other issues	Dropping out of school in young adolescence, legitimacy of an artificial abortion, overprotective parents, and barriers of parent-child communication were found. (Patient backgrounds: Young adolescents, misconduct, dropping out of school) In addition to biological factors, psychosocial issues should also be addressed. (Patient backgrounds: Overprotective mother)
Deeply discussing psychosocial issues	Facilitating group dynamic on issues	Regarding a health care delivery system, everyone should express his or her opinions on how to change our national health insurance system. (The medical ecological system: Deteriorating (unfriendly) medical practice environment)
Deeply discussing action plans	Facilitate group dynamics on action plan	You must evaluate and reflect on whether you have the ability to educate a young adolescent female to be adequately competent for safe sexual behavior. Did you receive sufficient training? Where can you find study materials? (Patient’ backgrounds: Young adolescent misconduct, school dropout)
Explore new knowledge about psychosocial issues	Sharing knowledge fact Suggest further reading materials Explore and facilitate the discussion to cultural perspectives	I would like to share a book entitled *Doctors’ Social Responsibility*. The main message is that a doctor not only treats patients in the hospital. Some conduct research, are involved in medical education, or advocate a public health policy. This book described how doctors can become involved in social situations. (The medical ecological system: Rural medical care system) Western medicine is individualistically oriented; therefore, patient autonomy is crucial. By contrast, our culture is familial, in which the family's role is consequential in nearly everything. Therefore, it is difficult to promote DNR when family members obstruct medical personnel to reveal a patient's condition to the patient. Sometimes we introduce something that may not be suitable to our society and must be modified. (Medical communication: Informing vs. concealing the illness situation from the patient and family members)
Explore new knowledge about action plan	Point out the diversity of human nature Point out the important of interpersonal and communication skills	You are facing people from diverse families and backgrounds. Therefore, my suggestion for your action plan is to learn more from various people and talk to them to learn their individual differences. Medical students’ growth environments are relatively simple. Your communication skills must be improved and you must have diverse friends. (Grandparenting)
Encouraging or approving students	Echoing the same experiences Positive feedback to medical students	That's right! My experience was the same. It is good that you feel this way, which is consistent with the feedback. We can all learn from it. (Medical communication: Drug compliance)
